# Hiccups: A new explanation for the mysterious reflex

**DOI:** 10.1002/bies.201100194

**Published:** 2012-02-29

**Authors:** Daniel Howes

**Affiliations:** Queen's University Department of Emergency MedicineKingston, Ontario, Canada

**Keywords:** hiccup

The common hiccup is a ubiquitous reflex; everyone experiences hiccups innumerable times through life, but unlike the other common reflexes like sneezing (clearing material from the nasal passages) and coughing (clearing material from the airways) there is no known physiologic advantage for the common hiccup 1–4.

Rather than continuing as a vestigial reflex whose purpose has evolved away, I propose that the hiccup may be a surprisingly complex reflex to remove air from the stomachs of young suckling mammals.

The hiccup (or hiccough) is an onomatopoeic name that comes from the sound made by the abrupt closure of the vocal cords approximately 35 milliseconds after the forceful contraction of the respiratory muscles. In the medical literature, hiccups are referred to as ‘singultus’, although this term was originally used to describe the sharp intake of breath often associated with long periods of crying.

When hiccups continue for more than 48 hours or occur frequently they may be a sign of a serious disease. More often they go unnoticed or are considered a minor annoyance that serves no valuable purpose.

Hiccups seem to occur in most mammals. They have been studied in cats, rats, and rabbits 1, 5, and are often observed in horses, dogs, and humans. The rhythmic movement of hiccups can be felt by pregnant mothers and seen on ultrasound occurring in the fetus in utero, before swallowing or respiratory reflexes appear. The reflex is most prevalent in newborns and they spend as much as 2.5% of their time hiccupping 2, it then diminishes in infancy with occasional brief recurrences through life 1. There do not appear to be documented observations of hiccups in reptiles, amphibians, or birds.

Much of what is known about the anatomy of the hiccup reflex comes from the study of pathological hiccups, which can arise as a result of infection or malignancy near the diaphragm, or from lesions in the brain. Afferent signals come from the distal esophagus, stomach, and the abdominal side of the diaphragm and travel as part of the phrenic nerve, the vagus, and sympathetic (T6-T12) chain branches. The afferent limb path is variable between individuals, as is the degree of stimulus required to initiate the reflex.

The central component of the reflex lies in the medulla. Electrophysiological studies as well as the pattern of muscle contraction suggest that the center for the hiccup reflex is entirely separate from the pathways involved in rhythmic breathing 6. A series of patients with lateral medullary infarction (Wallenberg's syndrome) and hiccups suggest that middle level and dorsolateral lesions can induce hiccups.

Once initiated, hiccups usually occur at a rate of 4–60 per minute. The frequency remains fairly constant in the individual, but can be modified by various conditions. Hiccups are suppressed by elevations in serum carbon dioxide 3 and can be triggered by gastric distention, rapid eating, or drinking carbonated beverages 1.

Efferent nerves travel from the hiccup center to the diaphragm, the external intercostals, the scalene muscles, glottic structures, and the esophagus. The most significant muscle group involved is the diaphragm, and several studies have shown that hiccups are often unilateral, involving only the left hemi-diaphragm 7, 8.

The result is the activation of the respiratory muscles more vigorously than with normal respiration, followed approximately 35 milliseconds later by closure of the glottis 3. This forceful inhalation effort against the closed glottis leads to a sharp reduction in intra-thoracic pressure. At the same time, normal esophageal peristalsis is suppressed and the lower-esophageal sphincter relaxes. Innervation to the muscles of exhalation is inhibited.

The drop in intra-thoracic pressure is experienced by the lungs, the heart, and great vessels, lymphatic vessels, the thymus, and the esophagus where it traverses the thorax. The stomach lies underneath the diaphragm and is outside the thoracic cavity.

## Previous theories of why we hiccup

A hypothesis seeking to explain the purpose of the hiccup should meet the following criteria in order to be considered plausible:

1. The hypothetical stimulus of the hiccup should be anatomically consistent with the afferent limb of the reflex.
2. The activation of the efferent limb of the reflex should resolve or help to resolve the condition that leads to the stimulus. Ideally the condition should explain all of the components of the efferent limb.
3. The hypothesis should offer an explanation for the hiccup's prevalence in mammals and its profoundly increased incidence during infancy.
4. The resolution of the condition that is hypothesized to stimulate the hiccup should offer a tangible evolutionary advantage.

Many purposes have been proposed for the hiccup, but to date none has met all four of the criteria above.

Suggestions that hiccup represent a form of epilepsy 9 or a failure of supraspinal inhibition 10 may suggest causes for pathological hiccups, but they do not explain the presence of the reflex in the normal individual. Similarly, the suggestion that the hiccup could be a dysfunction of the reciprocal inhibition of an inspiratory effort related to breathing and a simultaneous glottic closure related to swallowing 11 fails to explain the afferent limb of the reflex. Furthermore, hiccups are often present in the absence of swallowing.

In 1899, Ferroni suggested that hiccups were a form of preparation for the fetus to strengthen the muscles involved in respiration, a hypothesis more recently revisited by Kahrilas and Shi 2. The respiratory exercise hypothesis does not explain the existence of the afferent limb of the reflex. Furthermore, the brief contraction of the respiratory muscles is unlikely to have any beneficial effect on respiratory muscles, which have a high concentration of slow twitch fibers for endurance.

Other suggestions related to fetal development have included clearance of meconium (the first feces that a newborn produces; in times of fetal distress meconium can be passed while in the uterus and then breathed in) and training for suckling, but these do not seem plausible in light of the actions of the reflex. The strong contraction of the respiratory muscles would move meconium deeper into the airway, and the majority of the muscles triggered by the hiccup reflex are not involved in suckling.

Straus et al. 1 proposed a phylogenic hypothesis that the hiccup is an evolutionary remnant that originated with gill ventilation. They make an excellent argument for the phylogenic development of the hiccup reflex from ventilatory motor patterns of lower vertebrates and suggest that the hiccup is an evolutionary remnant. This hypothesis – that there is no purpose for the hiccup – should only be accepted in the absence of an acceptable explanation for its evolutionary persistence.

Others have suggested that the hiccup is a reflex to move boluses of food trapped in the esophagus 4. This theory is supported by the afferent innervation of the reflex, suggesting that the stimulus is a condition sensed in the area of the lower esophagus, stomach, or beneath the diaphragm. It would also explain the simultaneous relaxation of the lower-esophageal sphincter. The main problem with this theory is that the action of the hiccup would move a food bolus toward the middle of the chest – a food bolus in the lower esophagus would move away from the stomach where it can be safely digested and toward the airway, where it could become a dangerous obstruction. The fact that patients who present with food stuck in the esophagus rarely have associated hiccups and the high prevalence of hiccups in suckling newborns who do not consume solids, do not support this hypothesis.

## The most notable action of the hiccup reflex is the sharp drop in intra-thoracic pressure

The closure of the glottis with strong contraction of the respiratory muscles results in a sharp drop in the intra-thoracic pressure, which suggests that the purpose of this reflex is to move something from outside of the thoracic cavity toward the inside. There are five conduits between the intra- and extra-thoracic areas that contain fluid or air that might be moved, namely arterial, venous, and lymphatic vessels, the trachea, and the esophagus.

The arterial blood vessels have a high-pressure flow that would be expected to change very little as a result of the brief action of a hiccup. Venous and lymphatic flow may be somewhat increased by the action of the hiccup, but it is unlikely that it is significantly changed by such a brief stimulus. In addition to that, stimuli in the area of the afferent limb are unlikely to be resolved by the impact on blood or lymph flow.

A number of the previously offered explanations for the hiccup suggest the trachea as the conduit of interest, but this seems unlikely. The rapid closure of the glottis serves to prevent most of the air movement through the trachea, movement that could be much greater if the timing were different. Foreign material partially obstructing the airway would be moved deeper into the airway by a hiccup, thereby impeding clearance. Moreover, the airway is already well protected by gag, cough, and sneeze reflexes, the afferent limbs of which are more appropriately situated.

This leaves the esophagus. The negative intra-thoracic pressure of the hiccup would move material from the mouth or the stomach toward the mid-section of the esophagus. The existence of the swallowing reflex for moving material from the mouth, as well as the anatomy of the afferent limb, suggest the purpose of the hiccup relates to the lower esophagus instead of the upper esophagus or mouth.

The contents of the stomach are the materials of digestion and gas. The vomiting reflex effectively removes unwanted materials of digestion, leaving stomach gas as the potential trigger for physiologic hiccups.

## The hiccup as a burping reflex

**Figure 1:**
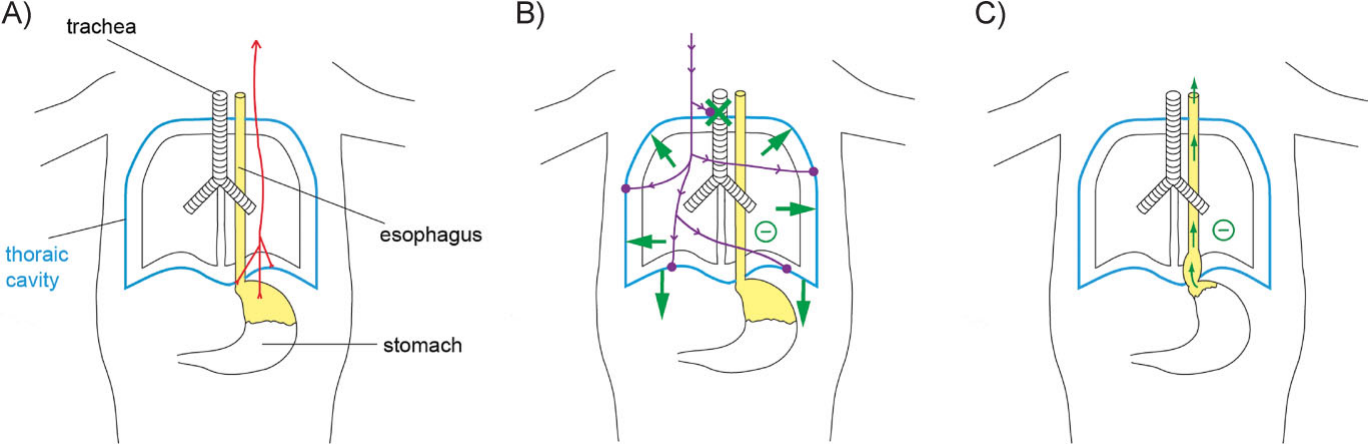
The hiccup may have evolved to remove air from the stomachs of young suckling mammals. **A**: The presence of air (yellow) in the stomach beneath the diaphragm triggers the afferent limb of the reflex, sending signals to the medulla (shown in red). **B**: Activation of the reflex efferent limb (purple) causes the muscles of respiration to expand the chest (green arrows), while simultaneously closing the opening of the trachea (green X). The result is a sharp drop in pressure in the chest (symbolized by 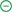). **C**: The negative intra-thoracic pressure moves the air bubble to the thoracic esophagus. With relaxation after the hiccup, the air can pass up the esophagus and out the mouth, leaving more room for milk.

Is it possible that the hiccup functions to remove swallowed gas from the stomach – essentially an evolved burping reflex? The presence of an air bubble in the stomach or distal esophagus could stimulate mechanoreceptors that activate the afferent limb of the reflex (Fig. 1A). The contraction of the respiratory muscles and closure of the glottis would drop the intra-thoracic pressure (Fig. 1B), pulling the air from the stomach in to the mid-esophagus (Fig. 1C), where it could then leave through the mouth with the next exhalation. This explanation is supported by the suppression of esophageal peristalsis and the relaxation of the lower esophageal sphincter that are part of the reflex.

The presence of a burping reflex provides a significant survival advantage. Young mammals depend on milk consumption for their nutrition. The continuous nature of suckling means that it has to be coordinated with respiration and the result can be swallowed air. A reflex that helps remove swallowed air would significantly increase the stomach's capacity for milk. This also explains why the hiccup is so much more frequent in infancy.

If this hypothesis is true, there must be something about the presence of air in the stomach that can be differentiated from distention with food. A future test of the hypothesis could be to identify how the reflex differentiates air; either directly or from a pattern of stimuli that occurs with the movement of an air bubble.

## Conclusion

The hiccup is a very common reflex. I propose that hiccups are triggered by the presence of air in the stomach. This stimulates the sharp intake typical of the reflex, moving swallowed air out of the stomach and effectively ‘burping’ suckling infants, allowing them to consume a greater volume of milk in the meal. For adults, the infrequent annoying affliction reflects persistence of an infantile reflex and a reminder that we may have eaten too quickly.

There is, as yet, no proof for this hypothesis, but hopefully it will stimulate some thought about this ubiquitous unexplained reflex, and provide a framework for exploring its anatomy and physiology in greater detail.

## References

[1] Straus C, Vasilakos K, Wilson RJA, Oshima T (2003). A phylogenetic hypothesis for the origin of hiccough. BioEssays.

[2] Kahrilas PJ, Shi G (1997). Why do we hiccup. Gut.

[3] Lewis JH (1985). Hiccups: causes and cures. J Clin Gastroenterol.

[4] Fass R, Higa L, Kodner A, Mayer EA (1997). Stimulus and site specific induction of hiccups in the oesophagus of normal subjects. Gut.

[5] Oshima T, Sakamoto M, Arita H (1994). Hiccup like response elicited by mechanical stimulation of dorsal epipharynx of cats. J Appl Physiol.

[6] Davis JN (1970). An experimental study of hiccup. Brain.

[7] Samuels L (1952). Hiccup: a ten year review of anatomy, etiology, and treatment. Can Med Assoc J.

[8] Salem MR, Baraka A, Rattenborg CC, Holaday DA (1967). Treatment of hiccups by pharyngeal stimulation in anesthetized and conscious subjects. JAMA.

[9] Launois S, Bizec JL, Whitelaw WA, Cabane J (1993). Hiccup in adults: an overview. Eur Respir J.

[10] McFarling DA, Susac JO (1979). Hoquet Diabolique: intractable hiccups as a manifestation of multiple sclerosis. Neurology.

[11] Askenasy JJ (1992). About the mechanism of hiccup. Eur Neurol.

